# Bioactive Levan-Type Exopolysaccharide Produced by *Pantoea agglomerans* ZMR7: Characterization and Optimization for Enhanced Production

**DOI:** 10.4014/jmb.2101.01025

**Published:** 2021-04-05

**Authors:** Safaa A. S. Al-Qaysi, Halah Al-Haideri, Sana M. Al-Shimmary, Jasim M. Abdulhameed, Othman I. Alajrawy, Mohammad M. Al-Halbosiy, Tarek A. A. Moussa, Mohamed G. Farahat

**Affiliations:** 1 Department of Biology, College of Science (for Women), University of Baghdad, Baghdad, Iraq; 2 Anbar Education Directors, Al-Anbar, Iraq; 3 Department of Applied Chemistry, College of Applied Science, University of Fallujah, Iraq; 4 Biotechnology Research Center, University of Al- Nahrain, Baghdad, Iraq; 5 Department of Botany and Microbiology, Faculty of Science, Cairo University, Giza 12613, Egypt; 6 Bionanotechnology Program, Faculty of Nanotechnology for Postgraduate Studies, Cairo University, Sheikh Zayed Branch Campus, Sheikh Zayed City, Giza 12588, Egypt

**Keywords:** Levan, exopolysaccharide, characterization, antioxidant, antiparasitic, antiproliferation

## Abstract

Levan is an industrially important, functional biopolymer with considerable applications in the food and pharmaceutical fields owing to its safety and biocompatibility. Here, levan-type exopolysaccharide produced by *Pantoea agglomerans* ZMR7 was purified by cold ethanol precipitation and characterized using TLC, FTIR, ^1^H, and ^13^C NMR spectroscopy. The maximum production of levan (28.4 g/l) was achieved when sucrose and ammonium chloride were used as carbon and nitrogen sources, respectively, at 35°C and an initial pH of 8.0. Some biomedical applications of levan like antitumor, antiparasitic, and antioxidant activities were investigated in vitro. The results revealed the ability of levan at different concentrations to decrease the viability of rhabdomyosarcoma and breast cancer cells compared with untreated cancer cells. Levan appeared also to have high antiparasitic activity against the promastigote of *Leishmania tropica*. Furthermore, levan had strong DPPH radical scavenging (antioxidant) activity. These findings suggest that levan produced by *P. agglomerans* ZMR7 can serve as a natural biopolymer candidate for the pharmaceutical and medical fields.

## Introduction

Levan is a homo-exopolysaccharide, composed of fructose molecules with a glucose residue linked by β-(2-6) glycosidic bonds at the end, and this backbone makes levan a unique biopolymer [1]. This biopolymer is produced from sucrose as a sole substrate by many types of microorganisms through the activity of a specific enzyme known as levansucrase (E.C. 2.4.1.10), and released as exopolysaccharides (EPS). Various bacterial genera such as *Zymomonas*, *Pseudomonas*, *Corynebacterium*, *Leuconostoc*, *Bacillus*, *Lactobacillus*, *Streptococcus*, *Gluconoacetobacter*, *Aerobacter*, *Brachybacterium*, and *Enterococcus* [2-5], and a few plant species are known to produce levan [6, 7]. Hitherto, more than one hundred bacterial genera were described as levan producers [2, 8]. Levansucrase is secreted by microorganisms into the extracellular environment particularly at low and neutral pH [9]. This enzyme works efficiently when sucrose is used as a sole carbon source and displays low activity with other types of sugars such as raffinose, mannose, fructose, glucose, and others [10]. Levan is considered as a water-soluble biopolymer that is also soluble in oil because it contains the β-(2,6) linkage. On the other hand, this biopolymer is insoluble in a wide range of organic solvents such as ethanol, methanol, acetone, isopropanol, toluene, etc. Besides being non-toxic and an ocular non-irritant, levan has some additional merits such as fair heat stability, with a melting-point temperature of 225°C [10]. Many reports demonstrated that levan is used in the medicinal and pharmaceutical industries as an antibacterial, antiviral, antiparasitic, antitumor, antioxidant, antiobesity, hypolipidemic, antidiabetic, immunostimulant and cosmeceutical agent [11-14]. Moreover, this biopolymer can be used in the food industries as a stabilizer, emulsifier, sweetener and thickener as well as in food packaging in levan-based film [15]. Levan is also used in the bread-making industry due to its ability to form a hydrocolloid microgel [16]. Additionally, recent studies suggested that levan can be applied in materials chemistry as it is used to strengthen aluminum bars and the binding of some plastics due to its excellent tensile properties [17, 18]. The broad-spectrum usage and global demand for levan by various industries demonstrate the importance of this biopolymer, which is also known for its versatility, low cost, and large-scale production capability [19]. The present work addresses the ability of *Pentoea agglomerans* strain ZMR7, isolated from rhizospheric soil of maize plants (*Zea mays*), to produce a levan-type EPS with promising antitumor, antiparasitic and antioxidant activities.

## Materials and Methods

### Isolation, Screening and Identification of EPS-Producing Bacteria

The rhizospheric soil samples of maize plants (*Z. mays*) were collected from five farms in Baghdad Governorate, Iraq. Ten grams of rhizosphere soil were suspended in 90 ml of sterilized peptone water (Oxoid, UK) and then the suspensions were serially diluted. Subsequently, 100 μl of adequate dilutions were spread on nutrient agar plates (Oxoid, UK) and incubated under aerobic conditions at 35°C. After incubation for 24-48 h, the growing bacterial colonies were picked up and cultured on modified levan-screening medium (1% tryptone, 0.5% yeast extract, 0.25% K_2_PO_4_, 0.5% NaCl, 20% sucrose and 1.5% agar) at 35°C for 48 h. The slimy mucoid colonies were selected as potential levan producers [20]. The potential levan-producing bacteria were identified by analysis of the 16S rRNA gene sequence amplified using the primer pair, 518F (5′-CCAGCAGCCGCGGTAAT-3′) and 1492R (5′-GGYTACCTTGTTACGACTT-3′) [21].

### Production and Isolation of EPS

Microbial EPS production was performed using production medium [per liter of distilled water (DW): sucrose, 200 g; yeast extract 2.5 g; MgSO_4_·7H_2_O, 1.0 g; K_2_HPO_4_, 5.5 g; (NH_4_)_2_SO_4_, 1.0 g; pH 7.2] [22]. Bacterial inocula were prepared in 250-ml Erlenmeyer conical flasks containing 50 ml of nutrient broth. One loopful of overnight bacterial culture was inoculated in nutrient broth and incubated in a shaking incubator (100 rpm) for 48 h at 35 ± 2°C. The production media were inoculated with 5% (v/v) of the inoculum (7 × 10^6^ CFU/ml) and incubated at 35± 2°C for 48 h with gentle agitation (100 rpm). After the incubation period, the culture was centrifuged for 10 min at 10,000 g and the cell-free supernatant was mixed with 2 volumes of ice-cold absolute ethanol and chilled at 4°C for 48 h. The precipitated EPS was harvested by centrifugation for 15 min at 15,000 g at 4°C and the pellets were re-dissolved in DW and deproteinized seven times with 1/5 volume of Sevag reagent (chloroform/n-butanol, 5:1 v/v)[23]. Thereafter, the aqueous layer containing the deproteinized EPS was collected, mixed with 2 volumes of ice-cold absolute ethanol and chilled at 4°C for 48 h. The re-precipitated EPS was collected by centrifugation for 15 min at 15,000 g at 4°C and the collected pellet was washed three times with 1.5 volumes of absolute cold ethanol. Afterwards, the pellets were dissolved in DW and dialyzed through a membrane with a 10-kDa cutoff against DW several times for three days, freeze-dried and weighed [5]. The EPS content was estimated by the hydrolysis of EPS samples in 0.1 M HCl for 1 h at 100°C and the content of EPS in the hydrolyzed solution was analyzed as D-fructose by the dinitrosalicylic acid method [24]. The obtained quantity of D-fructose was then divided by a factor of 1.11 to calculate the amount of levan. The strain designated ZMR7, exhibiting maximum levan production, was selected for further investigations [25, 26].

### Characterization of the EPS

Thin-layer chromatography (TLC) of the acid-hydrolyzed biopolymer was conducted to investigate the composition of the EPS produced by *P. agglomerans* ZMR7. Briefly, the EPS was hydrolyzed using 5% HCL (v/v), then 2 μl of hydrolyzed EPS was spotted onto commercial, aluminum-backed TLC Silica Gel 60 F254 plates (Merck, Germany). Equivalent amounts of fructose, sucrose, and glucose were dissolved in 1 ml of 1% ethanol and used as standards. The plates were placed in a closed jar with mobile phase chloroform/acetic acid/water at a ratio (7:7:1). After drying, the plates were sprayed with a solution of H_2_SO_4_/ethanol (9:1 v/v) followed by heating for 5-10 min at 90°C to visualize the carbohydrates [27]. Then, the Rf values of the ascending materials were calculated [28].

The presence of fructose in the EPS produced by *P. agglomerans* ZMR7 was detected by potassium ferricyanide (PF) sugar test. Briefly, 1 ml PF solution (10 mg/ml in 20% NaOH) was added to 1 ml acid-hydrolyzed EPS (1 mg/ml), gently mixed, and the color change was observed. Fructose and glucose served as control [29]. The functional groups of the intact EPS produced by *P. agglomerans* ZMR7 were analyzed using a Shimadzu FT-IR-8300/8700 spectrometer (Shimadzu, Japan) between the 4,000-to-400 cm^-1^ region at mid-temperature (26 ± 1ºC), with a resolution of 4.0 cm^-1^ [25]. ^1^H and ^13^C nuclear magnetic resonance (NMR) spectra were measured and recorded using an Avance III 400 MHz spectrometer (Bruker, Germany). Ten milligrams of produced EPS were dissolved in 1 ml of deuterium oxide (D_2_O). The ^1^H NMR spectrum was run at 400 MHz while the ^13^C NMR spectrum was run at 100 MHz. The chemical shifts were obtained and presented as ppm. The obtained spectra were compared with those of other levans described in previous publications.

### Optimization of Levan Production

The optimization of cultural parameters was carried out by determining the effect of individual factors and incorporating these at the optimum level before optimizing the next parameter and the quantity of the produced levan was estimated. The effect of sucrose concentration on levan production was assessed at various concentrations (50, 100, 200, 300, 400, and 500 g/l). The production media were inoculated with *P. agglomerans* ZMR7 and incubated at 35°C for 48 h. Furthermore, six nitrogen sources were used to investigate their impact on levan production. The yeast extract in the original production medium was substituted uniquely with each nitrogen source (casein, peptone, yeast extract, beef extract, ammonium chloride and potassium nitrate). The optimum temperature for levan production was determined by cultivating *P. agglomerans* ZMR7 at various temperatures (25, 30, 35, 37, and 40oC). To assess the influence of pH value on the levan production by *P. agglomerans* ZMR7, the production medium was adjusted at various pH values (4.0, 5.0, 6.0, 7.0 and 8.0). The optimal incubation period for levan production was investigated by the determination of levan yield after different incubation periods (24, 48, 72, 96, and 120 h).

### Biological Activities of Levan Produced by *P. agglomerans* ZMR7

**Antiproliferation activity.** The antiproliferative activity of the produced levan was evaluated, in vitro, against rhabdomyosarcoma (RD) and breast cancer (MDA-MB-231 ATCC HTB-26) cell lines using colorimetric cell viability MTT assay [30]. Briefly, 100 μl/well of RD and MDR cells (10^6^ cell/ml) were cultured in RPMI-1640 medium supplemented with FBS (10%), penicillin (100 U/ml) and streptomycin (100 μg/ml) and added to 96-well tissue culture plates (50 μl/well). Then, 100 μl of *P. agglomerans* ZMR7 levan EPS solution was added to each well to final concentrations of 19, 39, 78,156, 312, 625, 1,250, 2,500 μg/ml and incubated at 37°C for 24 h. After incubation, 10 μl (3-(4, 5-dimethylthiazolyl-2)-2,5-diphenyltetrazolium bromide (MTT) solution (5 mg/ml) was added to each well and incubated at 37°C for 4 h. Finally, 50 μl dimethyl sulfoxide (DMSO) was added to each well and incubated for 10 min. RD and MDA cells cultured in the same medium without levan were used as control. The optical density (OD) was measured for each well at A_620_ nm using a microplate reader. The growth inhibition percent was calculated according to the formula:

$$\mathrm{Growth}\mathrm{Inhibition}(\mathrm{GI}\%)=\frac{\left(\mathrm{OD}\mathrm{of}\mathrm{control}\mathrm{wells–OD}\mathrm{of}\mathrm{test}\mathrm{wells}\right)}{\mathrm{OD}\mathrm{of}\mathrm{control}\mathrm{wells}}\times 100$$

**Antileishmanial activity.** The antileishmanial activity of levan produced by *P. agglomerans* ZMR7 against promastigote forms of *Leishmania tropica* was estimated by using the colorimetric cell viability MTT assay [31]. *Leishmania* promastigotes (100 μl/well) were cultured in 96-well tissue culture plates (10^6^ parasite/ml). Then, 100 μl of various concentrations of the produced levan EPS was added to each well to final concentrations of 19, 39, 78,156, 312, 625, 1,250, 2,500 μg/ml and incubated at 26°C for 24 h. After incubation, 10 μl of MTT solution (5 mg/ml) was added to each well and incubated at 26°C for 4 h. Finally, 50 μl DMSO was added to each well and incubated for another 10 min. Promastigotes cultured in the same medium without levan served as a control. The optical density (OD) was measured for each well at 620 nm using a microplate reader. The growth inhibition percent was calculated as follows:

$$\mathrm{Growth}\mathrm{Inhibition}(\mathrm{GI}\%)=\frac{\left(\mathrm{OD}\mathrm{of}\mathrm{control}\mathrm{wells}\mathrm{–OD}\mathrm{of}\mathrm{test}\mathrm{wells}\right)}{\mathrm{OD}\mathrm{of}\mathrm{control}\mathrm{wells}}\times 100$$

**Antioxidant Activity.** The antioxidant activity of the levan produced by *P. agglomerans* ZMR7 was determined in vitro using 2,2-diphenyl-1-picrylhydrazyl (DPPH) free radical scavenging assay. Levan solutions of different concentrations (0.2-2 mg/ml) were prepared in DW. One milliliter of levan EPS solution was intensively mixed well with 2 ml of freshly prepared DPPH radical solution and incubated in dark for 1 h at room temperature. Subsequently, the DPPH radical scavenging activity was determined by measuring the absorbance at A_517_ nm using the CE1021 UV-Visible Spectrophotometer (Cecil, UK). Levan solution in ethanol was used as a blank while a mixture of ethanol and DPPH was used as control. The DPPH scavenging inhibition % activity was calculated according to the following formula:

Scavengingactivity%={(B-S)/B}×100,

whereas, B (absorbance of blank), S (absorbance of the sample).

### Statistical Analysis

The statistical analysis was conducted by IBM SPSS Statistics software 22 for Windows (IBM, USA) and GraphPad Prism version 7 (GraphPad Software, Inc., USA). All data were expressed as mean ± SD and statistical analysis was performed by one-way analysis of variance (ANOVA) of Duncan’s test and *p* < 0.05 was recognized as significant.

## Results

### Isolation, Screening and Identification of EPS-Producing Bacteria

**Fig. 1:**
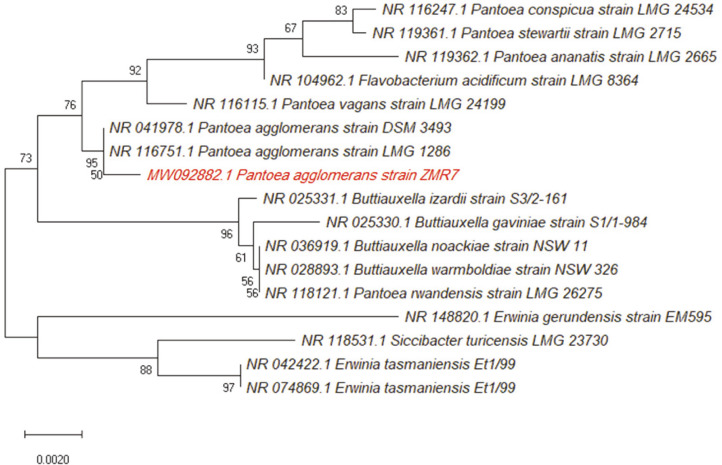
Neighbor-joining phylogenetic dendrogram based on 16S rDNA sequences shows the relationship between *P. agglomerans* ZMR7 and the related taxa.

A total of 128 bacterial colonies of different shapes were isolated from rhizospheres of maize plants. Of these isolates, 16 strains exhibited mucoid and slimy colonies on sucrose-rich media, which could be an indication for levan production. ESP-producing candidates were picked and further examined by quantitative estimation of the produced EPS. Based on the result of quantitative screening for EPS production, the most promising strain, designated ZMR7 and exhibiting the maximum production of the EPS (9.35 g/l), was selected for further investigations. Based on 16S rRNA gene sequence analysis, the most efficient strain was identified as *P. agglomerans* ZMR7 (Fig. 1). The nucleotide sequence for the strain was deposited at GenBank (Accession No. MW092882).

### Characterization of EPS

To determine the monosaccharide composition, the EPS produced by *P. agglomerans* ZMR7 was exposed to acid hydrolysis process followed by TLC. Results revealed that the EPS produced by *P. agglomerans* ZMR7 is composed of fructose only. The Rf value of fructose standard was identical or so close to that of the acid-hydrolyzed EPS. The Rf value of the EPS produced by *P. agglomerans* ZMR7 was 0.44, which was closer to that of fructose (0.43). The addition of the PF solution to hydrolyzed EPS, glucose, and fructose, produced a greenish-yellow color solution. The color disappeared within 2 min in the case of the hydrolyzed EPS and fructose whereas the color disappeared after about 5 min in the case of glucose. These findings suggest that the polysaccharide isolated from *P. agglomerans* ZMR7 would consist of repeating units of fructose.

**Fig. 2:**
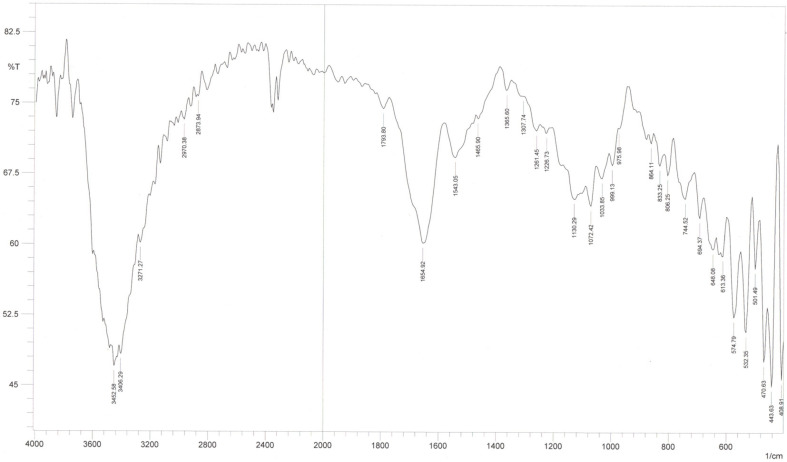
The FTIR spectrum of the levan produced by rhizospheric *P. agglomerans* ZMR7.

The chemical structure of the EPS produced by *P. aggloremans* ZMR7 was investigated by Fourier transform infrared spectroscopy (FTIR) spectroscopy (Fig. 2). The broad stretching peak of O-H stretching is present at 3,417 cm^-1^. The existence of C-H stretching vibration has been observed at around 2,935 cm^-1^. The three peaks appeared at 1,130, 1,083, and 1,045 cm^-1^ corresponding to the ring vibration overlapped with the stretching vibration of C-OH groups and the glycosidic linkage C-O-C stretching vibration. The FTIR spectroscopy data indicated that the biopolymer produced by *P. aggloremans* ZMR7 was levan type, as summarized in Table 1.

**Fig. 3:**
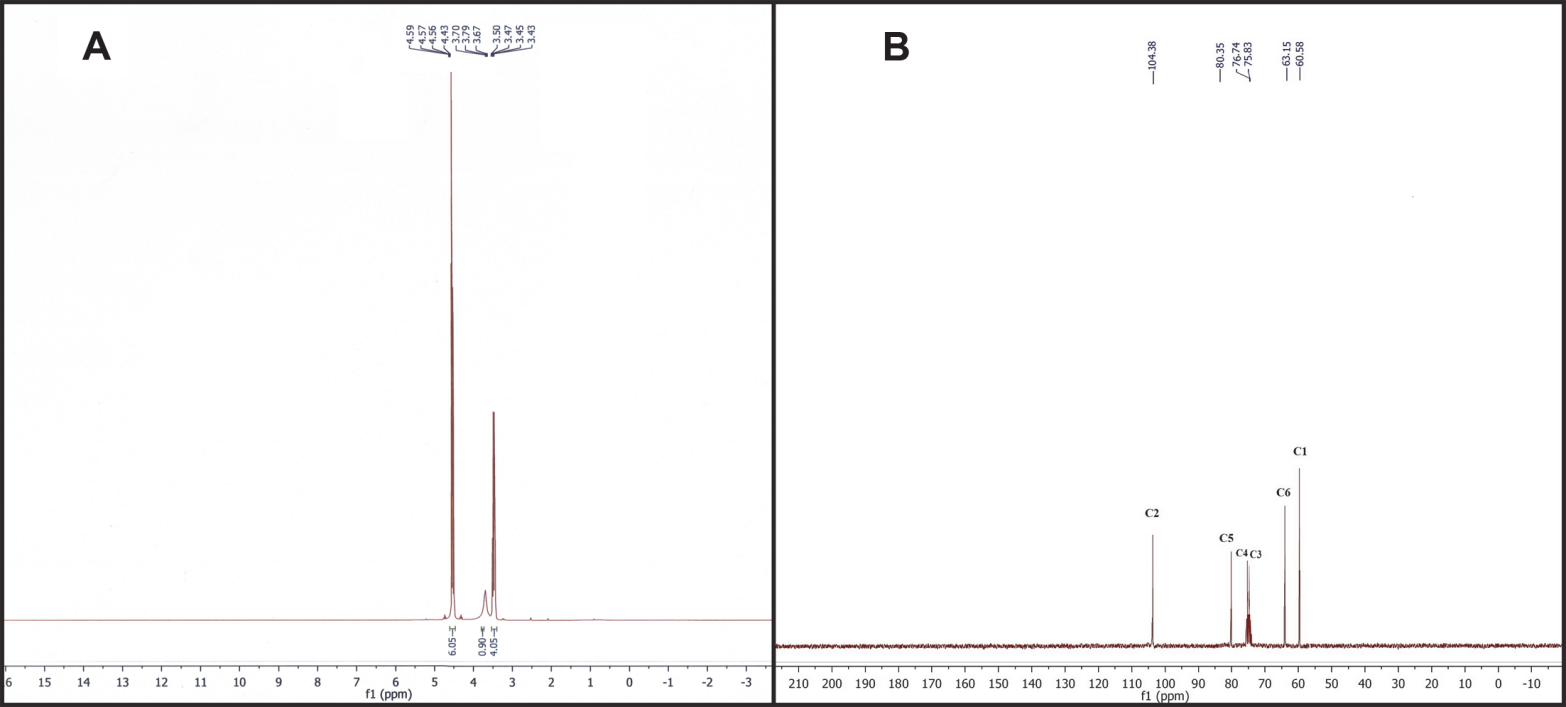
The ^1^H-NMR (A) and ^13^C-NMR spectra of the levan produced by *P. agglomerans* ZMR7.

The ^1^H-NMR spectrum of *P. agglomerans* ZMR7 EPS revealed characteristic signals of levan. All of the proton signals were observed between 3.43 and 4.59 ppm in the ^1^H-NMR spectrum. Noticeably, no peaks were observed in the anomeric region (5.3 to 4.6 ppm) (Fig. 3A), reflecting the absence of anomeric protons. Furthermore, the structure of the EPS produced by *P. agglomerans* ZMR7 was confirmed using ^13^C-NMR spectroscopy analysis. The ^13^C NMR spectrum showed six intense resonances at 60.58, 104.38, 75.83, 76.74, 80.35 and 63.15 that were assigned to the C1 to C6 atoms of the levan structure, respectively (Fig. 3B) (Table 2). It was noteworthy that the downfield signal shift at 63.15 ppm (C-6) revealed the β-(2→6) linkage. To the best of our knowledge, ours is the first report to describe and characterize the levan-type EPS from *Pantoea* species.

### Optimization of Levan Production

**Fig. 4:**
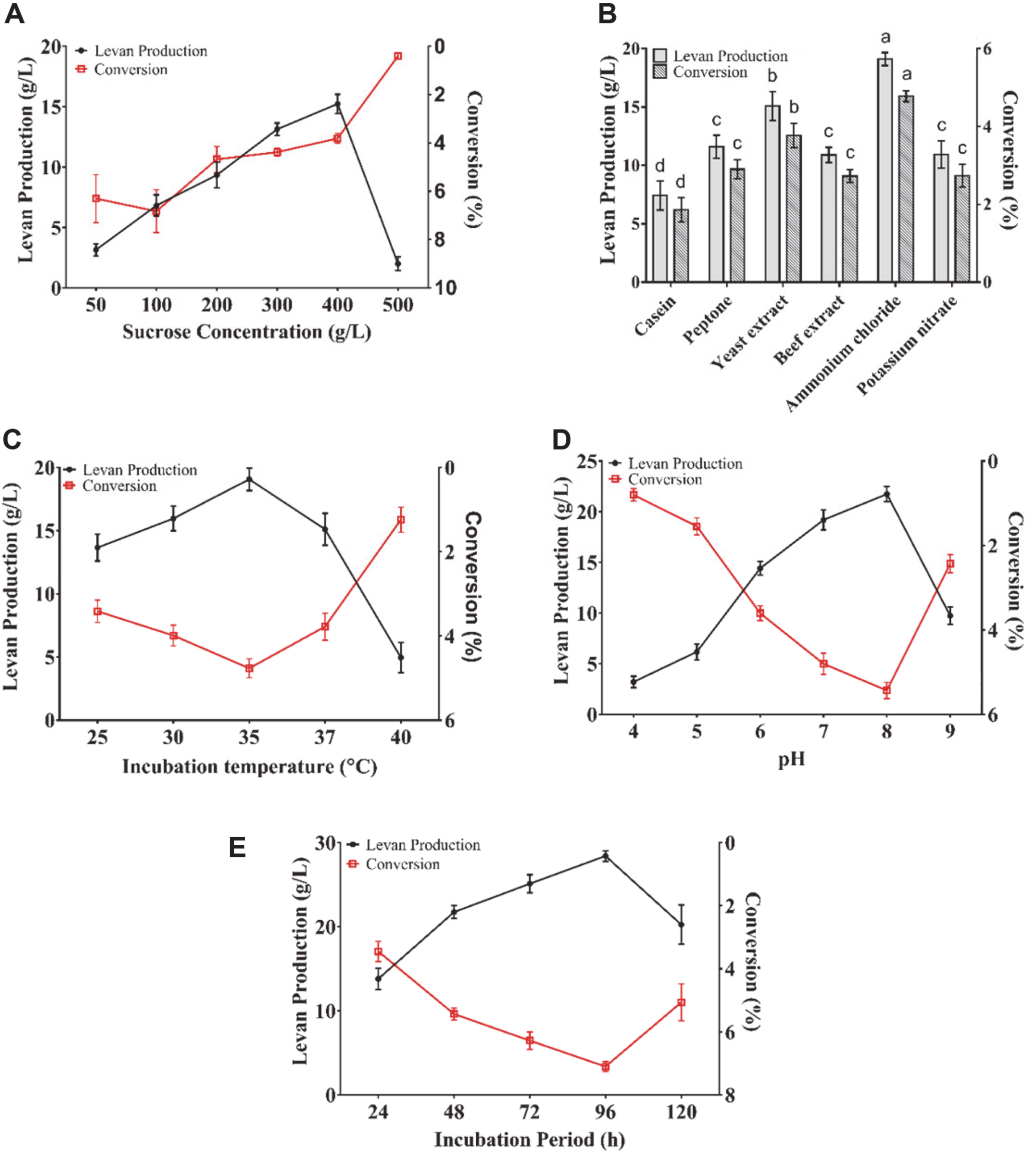
Impact of sucrose concentration (A), various nitrogen sources (B), incubation temperature (C), initial pH (D) and incubation period (E) on levan production and conversion of sucrose to levan by *P. agglomerans* ZMR7. Error bars represent standard deviations (SD). Columns headed by the same letter are not significantly different according to Duncan’s multiple range test (*p* < 0.05).

Unequivocally, results revealed the stimulating impact of increased sucrose concentration on levan production by *P. agglomerans* ZMR7 (Fig. 4A). The amount of produced levan was gradually increased by increasing the concentration of sucrose and the maximum amount of levan (15.23 g/l) was observed at a concentration of 400 g/l. Further increase in sucrose concentration resulted in a drastic decrease in the yield of levan. The levan yield was dramatically increased (19.11 g/l) when ammonium chloride was used as the sole nitrogen source, in comparison with the yield obtained by using yeast extract in the original production medium (Fig. 4B). The lowest yield of levan was observed when the production medium was supplemented with casein as a nitrogen source (7. 42 g/l).

To further investigate the optimal conditions of levan production, different incubation temperatures were evaluated. The results clarified that the optimal incubation temperature for levan production by *P. agglomerans* ZMR7 was 35°C at which the highest production level was observed. Notably, levan production is decreased when the temperature is raised over the optimal temperature (Fig. 4C). To precisely assess the effect of initial pH on the yield of levan, the production media were adjusted at various pH values ranged from 4.0 to 9.0. The amount of levan produced by *P. agglomerans* ZMR7 increased gradually with increase of the pH values from 4 to 8 (Fig. 4D). The highest amount of levan (21.74 g/l) was produced at a pH value of 8.0. The optimal incubation period for levan production was also investigated (Fig. 4E). The amount of produced levan progressively increased after 24 h of incubation time and the maximum levan production (28.4 g/l) was achieved after 96 h of incubation.

### Antiproliferative, Antileishmanial, and Antioxidant Activities of Levan Produced by *P. agglomerans* ZMR7

**Fig. 5:**
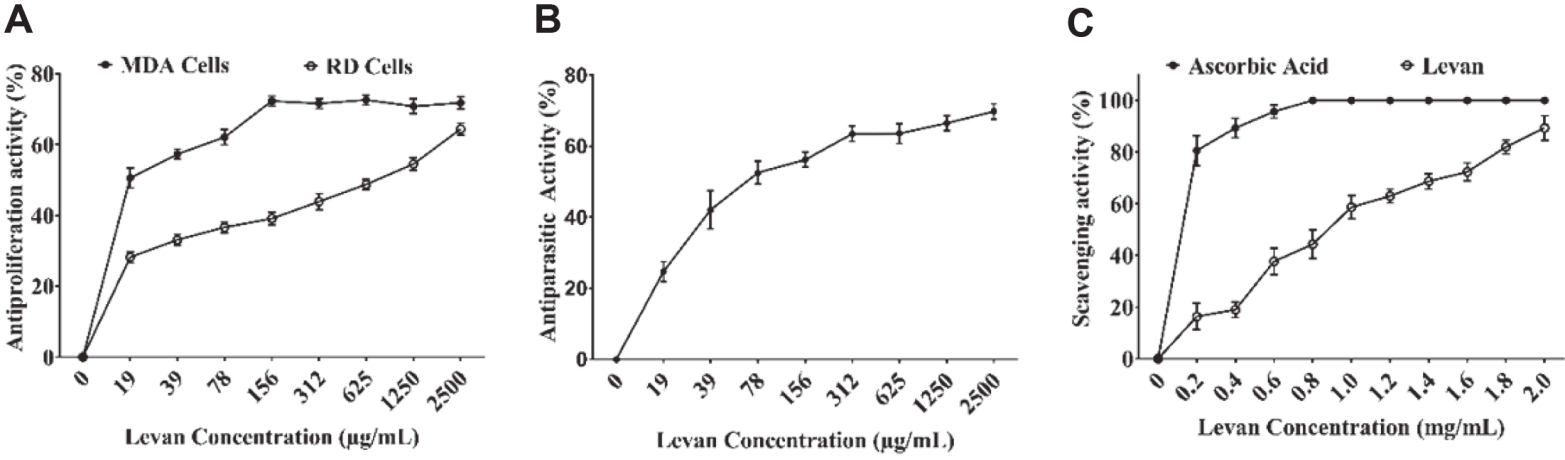
Biological activity of levan produced by *P. agglomerans* ZMR7: Antiproliferative effect against MDA and RD cell lines using MTT assay (A), antiparasitic effect against promastigotes of *L. tropica* (B), antioxidant activity (DPPH free radical scavenging ability) of levan produced by *P. agglomerans* ZMR7 and ascorbic acid as a standard (C).

Levan exhibited antiproliferative activities toward both investigated cell lines (Fig. 5A). The results revealed dose-dependent anticancer activities of levan against RD cells where the cytotoxicity effect increased by increasing the levan concentration. Notwithstanding, the levan produced by *P. agglomerans* ZMR7 showed different behavior towards MDA cells. The maximum cytotoxicity effect of levan (72.3%) was exerted at a concentration of 156 μg/mL and the further increase of the concentration did not promote further cytotoxicity. Consequently, the antiproliferative activity of levan could fluctuate towards different cell types. Levan produced by *P. agglomerans* ZMR7 reduced the viability of promastigotes of *L. tropica* at various concentrations (Fig. 5B). The growth of promastigotes of *L. tropica* treated with levan (2,500 μg/mL) was inhibited by about 69.8%, reflecting the potential antileishmanial activities of levan. Although the antileishmanial mechanism of levan is not known, it could be concluded that levan produced from *P. agglomerans* ZMR7 can be used as a putative antileishmanial agent.

To determine the antioxidant activity of levan produced by *P. agglomerans* ZMR7, DPPH free radical scavenging assay was used in comparison to ascorbic acid. The ascorbic acid served as a standard antioxidant agent at different concentrations. Ascorbic acid displayed about 80.6% scavenging activity at a concentration of 200 μg/ml, while the levan demonstrated 16.3% antioxidant activity at the same concentration (Fig. 5C). The highest antioxidant activity of levan was noticed at a concentration of 2 mg/ml, exhibiting 89.3% scavenging activity.

## Discussion

The newly isolated strain *P. agglomerans* ZMR7 exhibited promising ability to produce EPS from sucrose. In order to elucidate its structure, the purified EPS was subjected to FTIR, ^1^H NMR and ^13^C-NMR spectroscopy. Also, the monosaccharide composition of the purified EPS was investigated after the acid hydrolysis process. TLC analysis of the acid-hydrolyzed EPS clarified that fructose is the main composition of EPS. These results are similar to those of previous studies reporting the levan production by other bacterial strains [11, 27], suggesting the liberation of fructose only as a final hydrolysis product of the EPS. However, the Rf value for the same substance may differ according to various changes like different particle sizes for different batches of the adsorbent, the composition of the mobile phase, the amount of tank atmosphere saturation with mobile phase vapor, and storage conditions of silica gel plate [34]. The FTIR spectrum of the EPS produced by *P. aggloremans* ZMR7 shed light on its chemical structure. The stretching vibration of the existence of C-H and the broad band at 1,639 cm^-1^ were due to the bound water [32]. The observed three peaks corresponded to ring vibration overlapped with stretching vibration of C-OH groups and the glycosidic linkage C-O-C stretching vibration [29]. Besides, due to symmetrical stretching vibration, peaks appeared in the range of 990-806 cm^-1^, indicating the presence of the furanoid ring and the bending vibration of D-type C-H existing in furanose, respectively, which is typical of peaks of carbohydrates [34]. The FTIR spectrum result of the biopolymer produced by *P. aggloremans* ZMR7 in this study is consistent with FTIR spectra of levan produced by *Paenibacillus bovis*. nov BD3526 [32], *Bacillus megaterium* GJT321 [33], and *B. phenoliresistens* [4]. The levan-type EPS produced by *P. agglomerans* ZMR7 was also unveiled by ^1^H NMR spectroscopy, which revealed that the proton signals corresponded to the spectrum of the standard levan of *Zymomonas mobilis* [35], and *Bacillus licheniformis* levan [27]. This result was consistent with published data indicating that the ^1^H-NMR spectrum of a levan standard exhibits six proton shifts which were between (3.4 and 4.2) ppm [36]. Furthermore, ^1^HNMR data also clarified that the EPS produced by *P. agglomerans* ZMR7 was entirely constituted by fructose units, where peaks are missing in the anomeric region [37, 38]. ^13^C-NMR spectroscopy analysis has further confirmed the structure of the EPS produced by *P. agglomerans* ZMR7, and, the signal shifts also matched those of other levans produced by various bacteria such as *Z. mobilis* [25], *B. megaterium* GJT321 [33], and *Bacillus* sp. [14] isolated from honey. Additionally, the EPS is confirmed to be levan and not an inulin structure [39], where signals were shifted at 63.15 ppm.

Production medium development is an essential strategy to get a higher yield using any microbial strain [40]. Several factors affecting the production of levan were examined, with sucrose among the factors that enhanced the productivity of *P. agglomerans* ZMR7 for levan. The data in this study revealed that the increased concentrations of sucrose will inevitably increase the amount of levan production, whereas a noticeable decrease in the yield of levan was observed under a high concentration of sucrose. Taking this result, the amount of levan is proportional to sucrose concentration [41-43]. The stimulatory effect of sucrose could be because it serves as a substrate for levansucrase [37]. The observed adverse effect of sucrose at 500 g/l could be due to the inhibitory osmotic stress. These findings concur with the previous literature on levan [44]. In our study, ammonium chloride as the sole nitrogen source improved levan production in comparison with other investigated sources. Likewise, efficient levan production by *Bacillus licheniformis* NS032 using ammonium chloride as the sole nitrogen source has been reported [45]. However, several studies reported that the supplementation of organic nitrogen sources to the production medium for polysaccharide production could exert significant stimulating effects on the growth rate and the polysaccharide yield.

Interestingly, maximum levan production was detected at 35°C although the optimum temperature for growth of *P. agglomerans* ZMR7 is 30°C (data not shown). These findings suggest that levan production could be attributed to the optimum temperature for levansucrase activity rather than direct cell growth. Previously, several reports described the optimum temperature for the levan yield ranging from 25 to 37°C. Above this range of temperature, the levan yield may be reduced [46]. Several studies referred to the effect of acidity on levan production; here we intended to produce levan under different pH values. The result of our investigation revealed that *P. agglomerans* ZMR7 tends to produce levan in slightly alkaline conditions. On the other hand, the acidity was not shown to be suitable for good levan production and these results were consistent with those reported for levan production by *Halomonas smyrnensis* AAD6^T^, where the highest amount of levan was obtained at a pH ranged between 7-8 [47]. Regarding the incubation period, *P. agglomerans* ZMR7 produced the maximum amount of levan after 96 h of incubation. These results are consistent with previous results reporting the maximum production of levan after 72-120 h of incubation [4, 20]. It is worth mentioning that the overall optimization process enhanced the productivity of levan by *P. agglomerans* ZMR7. At optimum conditions, the strain ZMR7 converted 7.1% of sucrose to levan and remarkable productivity of the culture was gained (28.4 g/l). The optimized productivity of levan-type EPS attained in this work was higher than those obtained from *Bacillus tequilensis* GM (2.9 g/l) [48], *Bacillus subtilis* AF17 (7.9 g/l) [38], *Acetobacter xylinum* NCIM2526 (13.25 g/l) [29], and *Pseudomonas fluorescens* (15.42 g/l) [25]. Indeed, an efficient levan-producing *Pseudomonas mandelii* converted 37.8% of the sucrose in the medium to levan with a yield of 36.7 g/l [49]. Recently, it has been reported that Paenibacillus sp. strain FP01 produced levan with high yield (89.5 g/l) and was outstanding for its exceptionally high conversion rate of sucrose to levan (49.7%) [50].

The inhibitory activity of levan EPS produced by *P. agglomerans* ZMR7 towards RD cell lines is dose dependent. These results agree with previous studies reporting the inhibitory activity of levan against various cancer cell lines such as BGC-823 (human gastric cancer), A549 (human lung adenocarcinoma), HepG2/C3A (human liver hepatocellular carcinoma), AGS (human gastric adenocarcinoma), and MCF-7 (human breast adenocarcinoma)[51]. In contrast, the cytotoxic effect of levan EPS produced by *P. agglomerans* ZMR7 towards MDA was varied and does not correlate with levan concentrations. Consequently, the antiproliferative activity of levan could fluctuate towards different cell types. Nevertheless, the mode of action of levan against cancer cells is not well understood and it has been suggested that the inhibitory effects of levan towards MCF-7 cells were mediated by an increase in apoptosis and oxidative stress and associated with increased gene expression of p53 and p27 [52]. The antioxidant activity of levan EPS produced by *P. agglomerans* ZMR7 was examined and although it exhibits an antioxidant activity at only a high concentration in comparison to ascorbic acid, this result is in good agreement with previously published reports on the promising antioxidant activity of levan. The robust action of levan as an antioxidant agent may suggest that it is a good candidate antioxidant additive for pharmaceutical products, food processing, and other biomedical uses [29, 53].

## Conclusion

A new levan-producing strain of *P. agglomerans* ZMR7 was isolated from rhizospheric soil of *Z. mays* plant. The optimization process enhanced levan yield from 9.35 to 28.4 g/l. The levan production was stimulated by sucrose and ammonium chloride to produce an adequate amount of the biopolymer under optimum conditions. Moreover, levan produced by *P. agglomerans* ZMR7 exhibited promising anticancer, antiparasitic, and antioxidant activities, suggesting its potential application in the pharmaceutical field.

## Figures and Tables

**Table 1:** Comparison between FTIR values of levan produced by *P. agglomerans*, standard, and other bacteria.

Chemical Groups	Standard levan from *Z. mobilis* (cm^-1^)	*B. phenoliresistens* Levan (cm^-1^)	*P. bovis* sp. Levan (cm^-1^)	*B. megaterium* Levan (cm^-1^)	*P. agglomerans* Levan (cm^-1^)
O-H	3319.26	3394.1	3423	3354.37	3417.68
C-H	2935.48	2932.23	2935	2934.52	2935.66
C=O	1722.31	1647.88	1636	1647.93	1639.49

References	[25]	[4]	[32]	[33]	This study

**Table 2:** ^13^C-NMR chemical shift signal values of levan produced by *P. agglomerans* ZMR7 and other bacteria.

Carbon atom	^13^C-NMR chemical shift (ppm)			

	Standard levan from *Z. mobilis*	*B. megaterium* GJT321	*B. methylotrophicus* SK 21.002	*P. agglomerans* ZMR7
C-1	60.761	59.86	61.20	60.58
C-2	104.641	104.14	104.66	104.38
C-3	77.683	76.25	77.51	75.83
C-4	75.754	75.15	76.10	76.74
C-5	80.783	80.23	80.77	80.35
C-6	63.957	63.31	63.94	63.15

Reference	[25]	[33]	[23]	This study
